# Use of ATR-FTIR Spectroscopy and Chemometrics for the Variation of Active Components in Different Harvesting Periods of *Lonicera japonica*

**DOI:** 10.1155/2022/8850914

**Published:** 2022-03-07

**Authors:** Yun-Chu Zhang, Jing Deng, Xiu-Lian Lin, Ya-Mei Li, Han-Xuan Sheng, Bo-Hou Xia, Li-Mei Lin

**Affiliations:** ^1^Key Laboratory for Quality Evaluation of Bulk Herbs of Hunan Province, Hunan University of Chinese Medicine, Changsha 410208, China; ^2^State Key Laboratory of Natural Medicines (SKLNM) and Jiangsu Key Laboratory of Bioactive Natural Product Research, Department of Medicinal Chemistry, China Pharmaceutical University, Nanjing 210009, China; ^3^Hunan Key Laboratory of Druggability and Preparation Modification for Traditional Chinese Medicine, Changsha 410208, China

## Abstract

*Lonicera japonica* Thunb is a commonly used Chinese herbal medicine, which belongs to the family Caprifoliaceae. The active components varied greatly during bud development. Research on the variation of the main active components is significant for the timely harvesting and quality control of *Lonicera japonica*. In this study, the attenuated total reflection Fourier transform infrared spectroscopy (ATR-FTIR) combined with the chemometric method was performed to investigate the variability of different harvesting periods of *Lonicera japonica*. The preliminary characterization from ATR-FTIR fingerprints showed various characteristic absorption peaks of the main active components from the different harvesting times, such as flavonoids, organic acids, iridoids, and volatile oils. Additionally, principal component analysis (PCA) scatter plots showed that there was a clear clustering trend in the samples of the same harvesting period, and the samples of the different harvesting periods could be well distinguished. Finally, further analysis by the orthogonal partial least-squares discriminant analysis (OPLS-DA) showed that there were regular changes in flavonoids, phenolic acids, iridoids, and volatile oils in different harvesting periods. Therefore, ATR-FTIR, as a novel and convenient analytical method, could be applied to evaluate the quality of *Lonicera japonica*.

## 1. Introduction

Traditional Chinese medicine has a long history in China. It is a summary of the long-term life experience of the Chinese people. With the development of medical undertakings, Chinese medicines play an increasingly important role in disease treatments, beauty care, regimen, and immunity enhancement. The composition of Chinese medicines is complex and susceptible to the origin, climate, and harvesting periods, making it challenging to control quality. The quality of Chinese medicines is determined mainly by the content of active components, which is closely related to the harvesting period [1–4]. Therefore, the harvesting period is an essential factor affecting the quality of Chinese medicines.

*Lonicera japonica* is the dried flower buds, the initial opening flower, of *Lonicera japonica* Thunb. It has the efficacy of heat clearing, detoxifying, and evacuating wind heat [5]. The chemical composition of *Lonicera japonica* is complicated. It contains bioactive components, such as chlorogenic acid and its analogs, flavonoids, iridoid glycosides, and triterpenoid saponins [6]. At present, *Lonicera japonica* has been widely used in the medicine and food industry. However, the quality of Lonicera japonica on the market is mixed due to its concentrated flowering period, short harvesting time, and the effect of varieties, processing methods, and transportation conditions. Moreover, flower buds of different harvesting periods are easily mixed and distinctly different in the content of the main bioactive components, making it difficult to exert the corresponding clinical effect when used as drugs. Therefore, it is of great significance to study the variation of active components of *Lonicera japonica* in growth stages for its quality control.

Currently, in-depth research on the dissimilarity in different harvesting periods of *Lonicera japonica* is limited. The research on *Lonicera japonica* mainly focuses on the relevant aspects of the content changes in active components. Jiang et al. [7] studied the anti-inflammatory activity from different flowering phases of *Lonicera japonica* by high-performance liquid chromatography (HPLC). They found that the anti-inflammatory effects of Lonicera japonica gradually decrease as the flowering progressed. However, the preprocessing of this method is relatively complicated, and it acquires plenty of time and energy. Fu et al. [8] applied the HPLC to study the content changes in the primary antioxidants at different developmental stages of *Lonicera japonica*. The study revealed that chlorogenic acid and its derivatives have the strongest antioxidant capacity. Additionally, the highest content of chlorogenic acid was found before flowering. This helps to choose the harvesting period in which *Lonicera japonica* had the strongest antioxidant capacity. However, the shortcoming is that the flowering period is relatively short and not clear enough. Therefore, there is a need to develop a method and technique that is handy, rapid, economical, strong fingerprinting, and capable of fully characterizing the chemical composition of *Lonicera japonica*.

In addition to the above research methods, the common methods for studying the quality difference in Chinese medicinal materials in recent years include ultraviolet-visible spectrophotometry (UV-Vis), Fourier transform infrared spectroscopy (FTIR), liquid chromatography-mass spectrometer (LC-MS), gas chromatography-mass spectrometer (GC-MS), and nuclear magnetic resonance spectroscopy (NMR). Among them, UV-Vis has low sensitivity to compounds with weak UV absorption. The application scope of GC-MS is relatively limited, and it is often used to determine volatile compounds. The sample pretreatment and detection processes of LC-MS and NMR are complicated, and thus, they can be time-consuming and costly. However, FTIR technology is an effective, economical, fast, and strong fingerprinting detection method. It can be used nondestructively and rapidly to obtain biochemical fingerprints that provide information about molecular structure and composition [9]. Moreover, in recent years, ATR-FTIR technology has been developed based on FTIR technology, which can be equipped with ATR accessories for crystal materials, such as zinc selenide, germanium, and diamond. Only a small number of samples are needed, so sampling is quick and straightforward, with no pretreatment required. This technology is used for qualitative and quantitative analysis [10, 11]. It is a “green analysis method,” which has been extensively used in the research field of medicine and food chemical composition.

Therefore, in this study, the ATR-FTIR method was performed to establish the chemical information fingerprint of *Lonicera japonica* in five different harvesting periods. The sample data from these five periods were further analyzed and compared by applying chemometric methods, such as PCA and OPLS-DA. Moreover, to ensure the sustainable utilization of medicinal resources with high-quality and high-yield, the variation characteristics of the five periods were also discussed.

## 2. Materials and Methods

### 2.1. Instruments

The Fourier transform infrared spectrometer is equipped with ATR accessory (Nicolet iS5, Thermo Scientific, USA), 101 types of electrothermal blowing dry box (Beijing Yongguangming Medical Instrument Factory), DJ-10A pulverizer (Shanghai Longtuo Instrument Equipment Co., Ltd.), and 100 mesh standard sieve tray (Shangyu City, Zhejiang Province Yarn Screening Factory).

### 2.2. Plant Materials and Sample Collection

**Figure 1:**
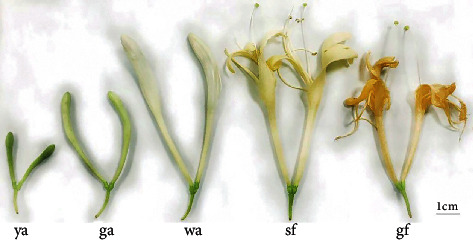
Sample characteristics of *Lonicera japonica* in five periods.

*Lonicera japonica* flower buds were collected from the medicine plantation of Hunan University of Chinese Medicine in April in Changsha, Hunan, China (112°54′E; 28°08′N). The well-developed *Lonicera japonica* samples were harvested at different time points. According to the color of the alabastrum, the flower bud differentiation process was divided into five periods including young alabastrum, green alabastrum, white alabastrum, silvery, and golden flower periods, named ya, ga, wa, sf, and gf, respectively (sample characteristics of each period are shown in Figure 1). The samples of three different positions were mixed in each period as one sample, and five samples were collected in each period.

### 2.3. Sample Preparation

The samples were rinsed with distilled water, were dried in the shade, and then placed in an oven. After drying at a constant temperature of 60°C until achieving constant weight, the samples were crushed and screened through a mesh size of 100. All samples were stored in a dryer until the ATR-FTIR analysis.

### 2.4. ATR-FTIR Spectra Collection

To deduct the interference of the absorption of dihydrogen oxide and carbon dioxide, the ATR-FTIR spectra of the air, accumulating 36 scans per spectra, were performed as background with 8 cm^−1^ resolution. When concerning sample test, a small amount of *Lonicera japonica* samples were taken on the dry and clean zinc selenide crystal material for spectral scanning in the range of 4000–500 cm^−1^ under the room temperature. After collecting every one sample, the ATR crystal was washed with a cotton ball dipped in alcohol until it was non-contaminated, and then, the next sample was collected.

### 2.5. Data Processing

Firstly, the ATR-FTIR fingerprint of *Lonicera japonica* was processed by Origin 2017 software, and then, SIMCA-P^+^ 15.0 software was adopted to conduct the first derivative (FD), second derivative (SD), standard normal variate (SNV), multiplicative scatter correction (MSC), Savitzky–Golay smoothing (S-G), exponentially weighted moving average (EWMA), and row center (RC) preprocessing on the spectral data. Each preprocessing method was processed by six scaling methods, namely unit variance (UV), unit variance none (UVN), Pareto (Par), Pareto none (ParN), centering (Ctr), and freeze (their definitions are shown in Table 1 [12, 13]). Then, the classification and discrimination models are constructed by combining PCA, OPLS-DA, and other chemometric methods. These preprocessing methods could improve the stability and accuracy of the model [14]. The optimal preprocessing method was SD. The *R*^2^ and *Q*^2^ values of the models range between 0 and 1, with higher values (greater than 0.5) indicating higher model fit and predictability [15]. Permutation tests (100 times) and S-line plots were used in the OPLS-DA model for model evaluation and screening of differential components. The *R*^2^*Y* (close to 1) and *Q*^2^*Y* intercepts (less than zero) in the permutation plot were applied to evaluate whether the model was overfitted [16]. S-line plots were used to identify statistically significant and potentially biochemically significant metabolites, based on both contributions to the model (greater than 0.8) and their reliability [17].

## 3. Results and Discussion

### 3.1. ATR-FTIR Fingerprint Analysis

**Figure 2:**
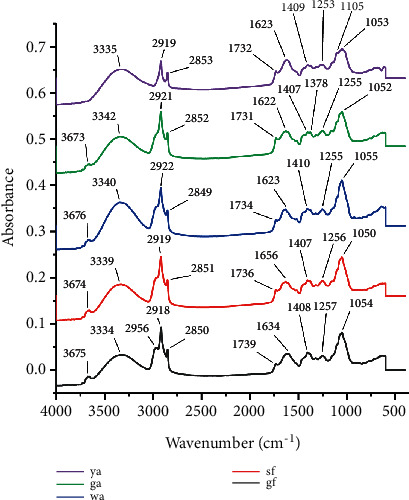
ATR-FTIR fingerprint of *Lonicera japonica* in different harvesting periods.

Infrared fingerprints have a large amount of information and strong specificity, which can provide the information of main chemical constituents in medicinal materials. Different chemical components have their own infrared characteristic peaks [18]. The difference in absorption value and peak intensity can reflect the dissimilarity of the main chemical components of *Lonicera japonica* in different periods. The ATR-FTIR fingerprint of different harvesting periods and the composition and group information of different bands are shown in Figure 2 and Table 2, respectively. The results showed that there was a sharp absorption peak appeared near 1105 cm^−1^ in the ya stage, which could be distinguished from other periods, corresponding to the overlapped absorption peak of -OH bending vibration and C-O stretching vibration of flavonoids or organic acids. The ga stage has obvious absorption near 1255 cm^−1^ and 1378 cm^−1^, corresponding to the characteristic absorption of C-O of organic acid. There was a strong infrared absorption peak near 1055 cm^−1^ at the wa stage, corresponding to the overlapped absorption peak of -OH bending vibration and C-O stretching vibration of phenolic acid or flavonoids. This indicated that the content of phenolic acids and flavonoids is abundant in this period. The sf stage has obvious absorption near 1656 cm^−1^, corresponding to the absorption peak of C=C stretching vibration of iridoids. At the gf stage, a sharp absorption peak near 2956 appeared, which could be distinguished from other periods, corresponding to the absorption peak of –CH_3_ stretching vibration of volatile oils. Additionally, there was an obvious absorption near 1634 cm^−1^, corresponding to the absorption peak of aromatic ring skeleton vibration of flavonoids or phenolic acid. It can be seen from the above analysis that the dissimilarity of these principal components might be an important reason for the variability of *Lonicera japonica* in different harvesting periods. However, due to the complexity of infrared fingerprints, it is difficult to distinguish the subtle differences in many bands by manual observation and comparison only; hence chemometric methods are desired to establish classification and discriminant models for further analysis.

### 3.2. Overall Variance Analysis by Principal Component Analysis

**Figure 3:**
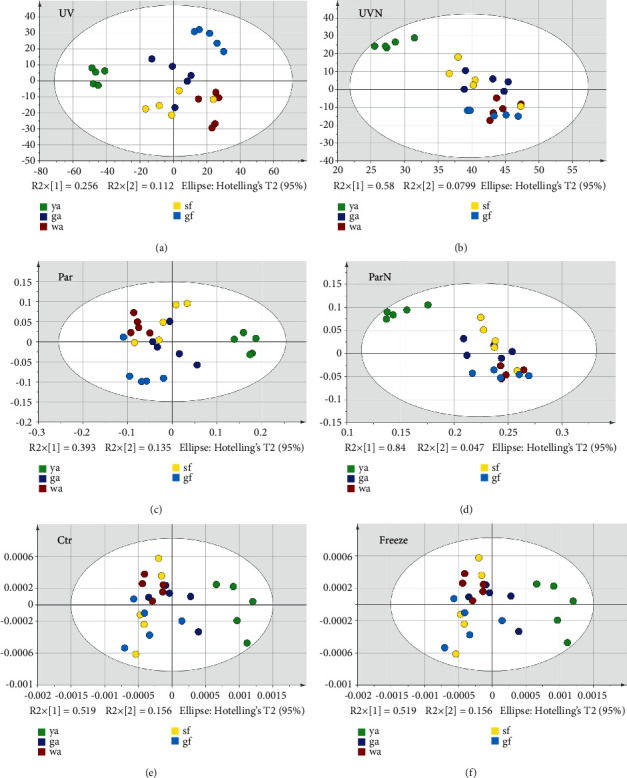
PCA scatter plot of six scaling methods.

**Figure 4:**
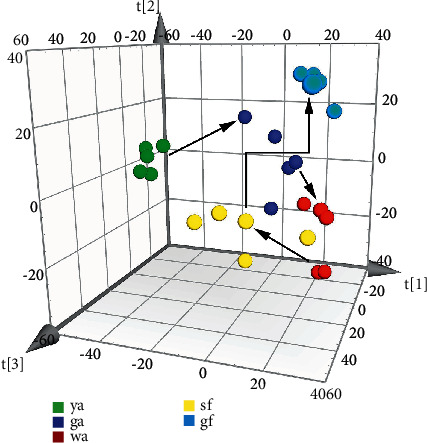
PCA scatter plot with UV scaling.

SIMCA is a supervised classification technique that uses samples with known origin (training samples) to derive a classification rule, which allows classifying new samples (test samples) with unknown origin in one of the classes, based on the values of the features of the new samples [34]. PCA is a mathematical algorithm that reduces the dimensionality of the data and can intuitively reflect the spatial distribution of the sample in the mathematical model while retaining most of the variation in the data set, which helps to understand the overall situation of the data and improve the accuracy of the model [35]. The results of the six scaling methods are shown in Figure 3. UV was chosen as the optimized scaling method for PCA model, and the results are shown in Figure 4 (three-dimensional). The model fitting parameter *R*^2^*X* = 0.784, and the model prediction parameter *Q*^2^ = 0.511, both of which are greater than 0.5, indicating that the model has a good fitting degree and strong predictive ability. There was a significant clustering trend of the *Lonicera japonica* samples in the same harvesting period. The samples in the ya stage and the gf stage could be significantly distinguished from those in other stages, indicating that the chemical components in these two periods are distinct. The spatial distribution of some samples in the ga stage, wa stage, and sf stage was relatively close, indicating that the variation of chemical components in these three periods was comparatively slight.

### 3.3. Individual Variance Analysis by Orthogonal Partial Least-Squares Discriminant Analysis

**Figure 5:**
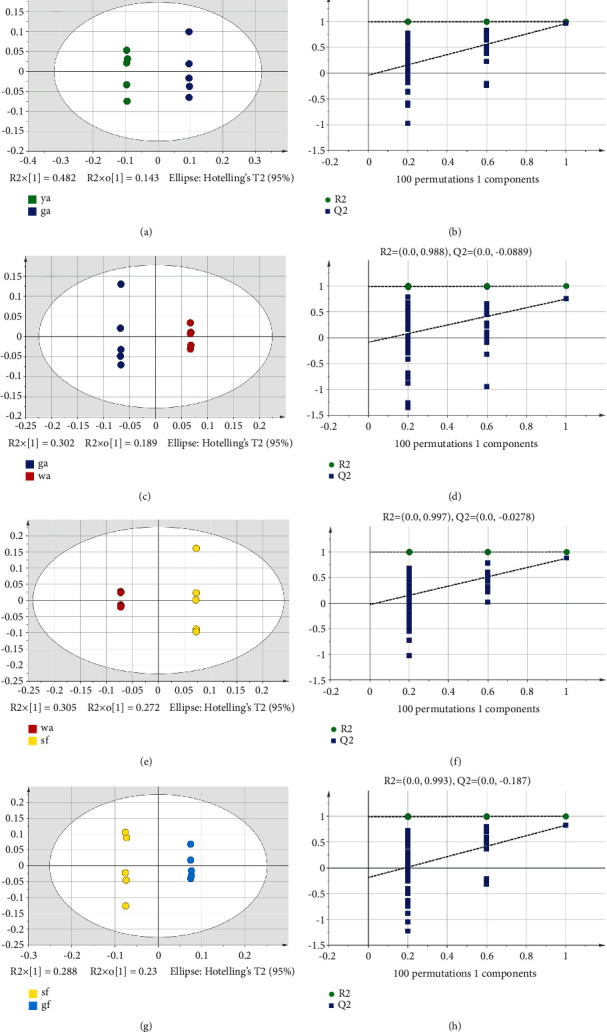
Scatter plot and permutation test plot of OPLS-DA models.

**Figure 6:**
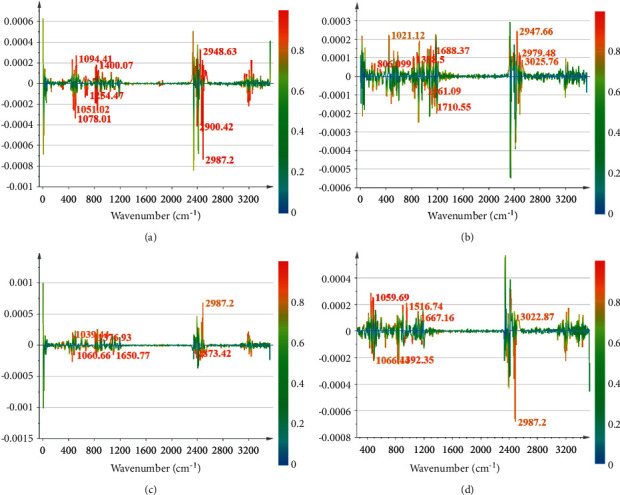
S-line plot of OPLS-DA models.

To better distinguish the differences between groups and to specify the dissimilarity between the two adjacent harvesting periods and their differential chemical compositions, the OPLS-DA was performed in this study [36]. The OPLS-DA scatter plot from the ya stage to the ga stage is shown in Figure 5(a). The model fitting parameter *R*^2^*Y* = *l*, and the model prediction parameter *Q*^2^ = 0.96, indicating that the model had a good fitting degree and strong predictive ability. It can be observed that the ya stage and the ga stage could be well distinguished, indicating that there were significant differences between the samples in these two periods. In the permutation test plot (Figure 5(b)), *R*^2^ = 0.999, *Q*^2^ = −0.0406, and all the points on the left of *R*^2^ and *Q*^2^ were lower than the rightmost point. It was concluded that the data were not overfitting, and the model could be used for the screening of differential markers. The contribution values greater than 0.8 in the S-line plot (Figure 6(a)) were 2987 cm^−1^, 2948 cm^−1^, 2900 cm^−1^, 1400 cm^−1^, 1254 cm^−1^, 1094 cm^−1^, 1078 cm^−1^, and 1051 cm^−1^. According to Table 1, it could be concluded that the main components that caused the change from the ya stage to the ga stage might be flavonoids or phenolic acid compounds, and nutrients, such as lipids, sugars, nucleic acids, and proteins. Research by Cui et al. [37] showed that the flavonoid content increased first and then decreased during the growth and development process of *Lonicera japonica* using the HPLC method. The increase in flavonoid content was probably caused by the increased activity of the chalcone isomerase, which catalyzes its synthesis [38]. Moreover, Zhang et al. [39] found that the content of phenolic acid compounds increased during the period from the ya stage to the ga stage. These research results were consistent with this study.

The OPLS-DA scatter plot from the ga stage to the wa stage is shown in Figure 5(c). The model fitting parameter *R*^2^*Y* = 1, and the model prediction parameter *Q*^2^ = 0.871, indicating that there was a good fitting degree and predictability. In the permutation test plot (Figure 5(d)), *R*^2^ = 0.988 and *Q*^2^ = −0.0889, and when the proportion of the displacing *Y* variable increases, the *Q*^2^ of the stochastic model gradually decreases, indicating that the data were not overfitting. According to the S-line plot (Figure 6(b)) based on the rule of the contribution values greater than 0.8, 3025 cm^−1^, 2979 cm^−1^, 2947 cm^−1^, 1710 cm^−1^, 1688 cm^−1^, and 1561 cm^−1^, 1388 cm^−1^, 1021 cm^−1^, and 806 cm^−1^ were confirmed. Accordingly, it could be concluded that the main components causing the change from the ga stage to the wa stage were similar to the result from the ya stage to the ga stage. However, among them, 1710 cm^−1^, 1688 cm^−1^, 1388 cm^−1^, and 806 cm^−1^ were the characteristic absorption of caffeoylquinic acid, chlorogenic acid, and flavonoids, which showed more chemical information of phenolic acids and flavonoids than other periods. Furthermore, the phenolic acids and flavonoids showed the most substantial infrared absorption peaks in the wa stage, indicating that the content of phenolic acids and flavonoids (the quality control index of *Lonicera japonica*) is at the highest level [40]. Meanwhile, Wang et al. [41] studied the content changes in the main active constituents of *Lonicera japonica* at different stages. The results showed that with the development of flower buds, the content of phenolic acids increased firstly, then decreased, and reached the peak value at the wa stage. Kong et al. [38] discovered that the content of flavonoids increased firstly and then decreased during the growth and development periods of *Lonicera japonica*. The highest content was also found at the wa stage. From the above study, phenolic acids and flavonoids were the differential components that caused the changes in the ga and wa stages, which were in line with the results of previous researchers.

The OPLS-DA scatter plot from the wa stage to the sf stage is shown in Figure 5(e). The model fitting parameter *R*^2^*Y* = 1, and the model prediction parameter *Q*^2^ = 0.919, indicating an excellent model fit and high predictability. In the permutation test plot (Figure 5(f)), *R*^2^ = 0.997 and *Q*^2^ = −0.0278, indicating that the model could be used for the screening of differential markers. The contribution values greater than 0.8 in the S-line plot (Figure 6(c)) were 2987 cm^−1^, 2873 cm^−1^, 1650 cm^−1^, 1376 cm^−1^, 1060 cm^−1^, and 1039 cm^−1^. It could be concluded that the main components causing the change from the wa stage to the sf stage might be iridoids, phenolic acids or flavonoids, and nutrients, such as lipids, sugars, nucleic acids, and proteins. Wang et al. [42] found that with the development of *Lonicera japonica* flower buds, the iridoids showed a trend, which decreased first, then increased, and finally dropped, with the highest content appearing at the wa stage and the sf stage. Fu et al. [8] observed that phenolic acid content was significantly reduced as the flowers opened. Therefore, phenolic acid content showed a downward trend from the wa stage to the sf stage. Cui et al. [37] revealed that the flavonoid content tends to decrease during the period from the wa stage to the sf stage. This might be attributed to the competition of chalcone isomerase for precursors at the sf stage [38]. Therefore, these results in this study were in agreement with previous reports.

Finally, the OPLS-DA scatter plot from the sf stage to the gf stage is shown in Figure 5(g). Similar to the above-established model, this model also showed a good fitting degree and strong predictive ability (*R*^2^*Y* = 0.999, *Q*^2^ = 0.827) and the permutation test plot (Figure 5(h)) showed nonsignificant overfitting (*R*^2^ = 0.993, *Q*^2^ = −0.187). According to the S-line plot (Figure 6(d)), the contribution values of 3022 cm^−1^, 2987 cm^−1^, 1667 cm^−1^, 1516 cm^−1^, 1392 cm^−1^, 1066 cm^−1^, and 1059 cm^−1^ were all greater than 0.8. This showed that the main components that caused the change from the sf stage to the gf stage might be volatile oils, phenolic acids or flavonoids, and nutrients, such as lipids, sugars, nucleic acids, and proteins. Wang et al. [41] showed that the content of volatile oil compounds increases in the flower bud stage, peaks in the sf stage, and then decreased. The change in volatile oil content showed certain flowering time dependence, indicating that some chemical components might accumulate during a particular period as the plant adapts to changes in its environment [43, 44]. Kong et al. [38] discovered that after the flowering of *Lonicera japonica*, the content of phenolic compounds significantly reduces till the gf stage when the content is minimal. The reduction in chlorogenic acid content might result from the increased biosynthesis of lignin using chlorogenic acid as a substrate during the gf stage. Zhang et al. [39] showed that the content of flavonoids decreases during the period from the sf stage to the gf stage of *Lonicera japonica*. These previous reports indicated that volatile oils, phenolic acids, and flavonoids were the differential components that cause changes in the sf and gf stages, which were in accordance with the results of this study.

In summary, ATR-FTIR combined with chemometrics was used to establish chemical information fingerprint and classification and discrimination model of *Lonicera japonica* in different harvesting periods. It showed that this method could scientifically evaluate the variation of active components among different harvesting periods of *Lonicera japonica*. Moreover, ATR-FTIR technology is simple, rapid, effective, and applicable to Chinese medicine. However, in the determination of the infrared spectrum, there exist some spectral absorptions of the interfering components and the absorption peak probably generates superposition phenomenon, which has a certain impact on the accuracy of the determination results. Therefore, the determination of the differential components of *Lonicera japonica* at different harvesting periods still needs to be further studied in conjunction with other analytical techniques.

## Figures and Tables

**Table 1:** Definition of six scaling methods.

Scaling method	Definition
UV	The variable *j* is centered and scaled to “unit variance,” which means that the base weight is computed as the reciprocal of the standard deviation of variable *j* computed around the mean (sdj)
UVN	Similar to UV, the variable is not centered, which means that the standard deviation is computed around zero
Par	Between no scaling and UV scaling. The variable *j* is centered and scaled to Pareto variance, which means that the base weight is computed as 1/sqrt (sdj)
ParN	Similar to par, however, the variable is not centered
Ctr	A column-wise centering to transform values varies around zero
Freeze	The scaling weight of the variable is frozen and will not be recomputed as observations in the work set change or the variable metric is modified

**Table 2:** Infrared spectrum band assignments.

Wavenumber (cm^−1^)	Vibration	Suggested biomolecular assignment	Reference
4000–3500	O-H stretching	H_2_O	[14]
3800–2600	-OH stretching	Water molecules	[19]
3615	*ν*_s_(OH) free	Water molecules	[19]
3490	*ν*_as_(OH), HB	Water molecules	[19]
3420, 3400	*ν*_s_(OH), HB	Water molecules	[19]
3400	O-H bond vibration	Sugar compound	[20]
3400	Free N-H stretching	Amide	[19]
3392	O-H stretching		[21]
3307	-OH deformation mode		[22]
∼3300	*ν*(NH), HB	Amide A, proteins	[19]
3286–3284	N-H stretching	Amide A of proteins	[23]
3286–3284	O-H stretching	Polysaccharides	[23]
3286	N-H stretching	Amide	[24]
3280	*ν*_s_(OH), HB	Water molecules	[19]
3280	H-O-H stretching		[25]
∼3100	*ν*(NH), HB	Amide B, proteins	[19]
3077	*ν*CH	Alkane/alkene	[24]
3030–2800		Lipids	[23]
3020–3010	*ν*(=CH)	Unsaturated fatty acids, cholesterol esters	[19]
3008	Olefinic HC=CH	Unsaturated lipids	[23]
3000–2800	-CH_3_ and -CH_2_ groups	Phospholipids and fatty acids	[19]
2960–2955	*ν*_as_(CH_3_)	Protein side chains, phospholipids, ceramides, fatty acids	[19]
2960	CH_3_- stretching (asym)	Alkane/alkene	[24]
2957	Asymmetric CH_3_ stretching		[25]
2956	CH_3_ antisymmetric stretching	Lipids, protein side chains	[23]
2924–2915	*ν*_as_(CH_2_)	Mainly lipids: phospholipids, ceramides, and fatty acids	[19]
2923, 2853	CH_2_- stretching		[21]
2923	CH_2_ antisymmetric stretching	Lipids (mainly)	[23]
2920	Asymmetric CH_2_ stretching		[25]
2900	C-H bonds	Sugar compounds	[20]
2875–2872	*ν*_s_(CH_3_)	Protein side chains, phospholipids, ceramides, fatty acids	[19]
2872	CH_3_- stretching (sym)	Alkane/alkene	[24]
2872	CH_3_ symmetric stretching	Proteins (mainly), lipids, carbohydrates, nucleic acids	[23]
2855–2847	*ν*_s_(CH_2_)	Mainly lipids: phospholipids, ceramides, fatty acids	[19]
2854	CH_2_ symmetric stretching	Lipids (mainly)	[26]
2844	CH_2_- stretching		[22]
2700–2330	NH^+^ stretching and overtones or combination bands in Fermi resonance	Tertiary amine hydrochloride salts	[9]
2500–2000		Unsaturated hydrocarbons	[27]
2442–2208	O-C-O stretching	CO_2_	[14]
2400–2250		CO_2_	[26]
2360, 2340		CO_2_	[26]
1900	The 2nd overtone of the CO bond	Caffeoylquinic acids	[28]
1800–1650		Chlorogenic acid	[29]
1800–900	Fingerprint region	All molecules	[23]
1742	Carbonyl C=O stretching	Cholesterol esters	[23]
1740		Triglycerides	[30]
1740–1720	*ν*(C=O)	Phospholipids, esters, glycerides	[19]
1734, 1627, 1522, 1440, 1410, 1367, 1315, 1255		Chlorogenic acid, flavonoids	[22]
1734	C=O stretching	Polyphenol	[22]
1720	The asymmetric overtone of the C-H bond	Caffeoylquinic acids	[28]
1700–1400		Fatty acids, lipids, proteins	[30]
1690–1610	*ν*(C=O)	Amide I (∼70–80% C=O stretch)	[19]
1682, 1639, 1471, 1284, 1181, 1111, 1032, 982, 950, 822, 789		Organic acids, flavonoids	[22]
1682	C=O stretching	Chlorogenic acid	[22]
1650	C=O stretching	Amide I (proteins, lipids, and carbohydrates)	[24]
1650	The first C-H overtone		[31]
1644		Triterpenoid saponin	[32]
1641	C=O stretching *ß*-sheet structure	Amide I (protein)	[23]
1636	C=C vibrations (aromatic ring skeletal)		[22]
1635		The crystalline water of sugar compounds	[20]
1627, 1522	C=C vibrations (aromatic ring skeletal)		[22]
1600–1450		Secologanic acid, chlorogenic acid, galuteolin	[33]
1590		Amino acids	[30]
1560–1500	*δ*(NH), *ν*(CN)	Amide II (∼40–60% N-H in-plane bend, ∼20–40% C-N stretch)	[19]
1545, 1455, 1450, 1410, 880, 875		Carbonate	[26]
1538	N-H bend, C-N stretch a helical structure	Amide II (protein)	[23]
1516		Tyrosine	[30]
1491	C=C aromatic ring stretching vibrations		[9]
1473–1468	*δ*(CH_2_), *δ*as(CH_3_)	Proteins, lipids	[19]
1462	*δ*(CH_2_), *δ*as(CH_3_)	Proteins, lipids	[19]
1454–1451	CH_2_ bending	Lipids	[23]
1454	*δ*(CH_2_), *δ*as(CH_3_)	Proteins, lipids	[19]
1453	CH_2_ scissoring		[25]
1440, 1410, 1376	OH vibrational modes	Organic acid	[22]
1428, 1407	NCH_3_ bending		[9]
1420–1300	The second overtone of the carbonyl group	Secologanic acid, chlorogenic acid, galuteolin	[33]
1413	C-H deformation vibration (CH_3_ and -CH_2_- groups)		[28]
1412	O-C=O symmetric stretching	Glycine	[25]
1400	*ν*(C=O) + *δ*s(CH_3_)	COO-: proteins, lipids, fatty acids	[19]
1398	CH_3_ bending	Proteins	[14]
1398	COO- stretching (sym)	Fatty acids, amino acids	[14]
1397	COO- symmetric stretching	Fatty acids	[23]
1394	C=O stretch of COO-		[25]
1393, 1358	*ν*CO/*ν*COO-, *δ*CHx (*x* = 1, 2, 3), *δ*NH vibrational modes	Proteins and lipids	[24]
1350	The second overtone of stretching O-H and C-H bonds		[31]
1343	*δ*(CH_2_)	C-H wagging	[19]
1333	C-H_2_ wagging	Glycine	[25]
1315, 1250	C-O stretching		[22]
1243, 1240	*ν*(CN), *δ*(NH) + *ν*_as_(PO_2_^−^)	Amide III (∼40% C-N stretch, 30% N-H in-plane bend, 20% methyl-C stretch)	[19]
1242	Asymmetric PO_2_^−^ stretch		[25]
1241	*ν*_as_(PO_2_^−^）	Nucleic acids, phospholipids	[30]
1234	PO_2_^−^ antisymmetric stretching	Nucleic acids (mainly), phospholipids	[23]
1200–1000	C-O stretching	Polysaccharides, glycosides	[21]
1200–1000		Nucleic acids, hydrocarbons, phosphates	[24]
1200–900	*ν*(CC), *δ*(COH)	Carbohydrates	[19]
1190–976, 962, 660–520, 472		Phosphate ions	[26]
1171	Ester C-O asymmetric stretch		[25]
1170–1100	The second overtone of C-H (-CH_2_)	Secologanic acid, chlorogenic acid, galuteolin	[33]
1167	C-H stretching (CH_3_ and -CH_2_- groups)		[28]
1166	CO-O-C antisymmetric stretching	Ester bonds in cholesteryl esters, ribose ring formations: RNA	[23]
1155	C-O stretching	Oligosaccharides, triacylglycerols	[14]
1152, 1080, 1022	*δ*(CHO), *ν*(C-O)	Glycogen, glucose	[24]
1150–850		Phosphate group	[26]
1150, 1020	-OH deformation modes		[22]
1141, 1079	C-O glycosidic bonds		[20]
1124	*ν*(C-O)	RNA	[30]
1120	C-O bonds	Ribose	[30]
1118	*ν*(C-O)	RNA, carbohydrates	[30]
1117	N-H_3_ rocking	Glycine	[25]
1091	p-Substituted aromatic vibrations		[9]
1090–1075	*ν*_s_(PO_2_^−^)	Phospholipids	[19]
1080	C-O stretch		[25]
1074	PO_2_^−^ symmetric stretching	Nucleic acids, phospholipids, glycogen, polysaccharides, glycolipids	[23]
1051	C-O stretching	Starch	[14]
1039	C-N stretching	Glycine	[25]
1034		Glycolytic components and nucleic acids	[24]
1010 (1225–950)	Aromatic C-H in-plane bending		[9]
996		Nucleotides	[19]
971	C-N^+^-C stretching	RNA	[23]
950–400		H_2_O	[24]
919	Ribose ring vibrations	RNA/DNA	[23]
914–600	O-C-O bending	CO_2_	[14]^1^
914	CH_2_ rocking	Glycine	[25]
894	The *ß*-configuration and *α*-configuration		[20]
877, 744		C-H deformation in proteins	[19]
833, 822	Aromatic C-H out-of-plane bending		[9]
771	C-O-C symmetry vibration	D-Glucopyranosyl ring	[20]
730–718	*ρ*(CH_2_)	Lipids	[19]

## Data Availability

All related data are included within the article.
